# Nematicide effects on non-target nematodes in bermudagrass

**DOI:** 10.21307/jofnem-2019-009

**Published:** 2019-04-15

**Authors:** Benjamin D. Waldo, Zane J. Grabau, Tesfamariam M. Mengistu, William T. Crow

**Affiliations:** 1 Entomology and Nematology Department, University of Florida, Gainesville, FL, 32611; 2 National Program Leader, Division of Plant Protection, National Institute of Food and Agriculture, Washington, DC, 20024

**Keywords:** Abamectin, Bermudagrass, *Cynodon* spp., Ecological indices, Ecology, Fluensulfone, Fluopyram, Furfural, Nematicides, Non-target nematodes, Turfgrass

## Abstract

In turfgrass systems, nematicides are a valuable tool for managing plant-parasitic nematode populations, but few studies have examined nematicide effects on non-target nematodes. The study evaluated effects of turfgrass nematicide formulations of abamectin (Divanem SC), fluopyram (Indemnify), furfural (MultiGuard Protect EC), and fluensulfone (Nimitz Pro G) on non-target nematode populations in bermudagrass (*Cynodon* spp.). Nematicides were applied at labeled rates every four weeks as a summer treatment program from June 7, 2016 to August 30, 2016 and April 24, 2017 to July 18, 2017. Samples were collected before the initial treatment and 2 d, 14 d, 56 d, and 238 d after the final treatment in both years for nematode community analysis. Data from each nematicide treatment were compared to the untreated at each sample date using analysis of covariance with initial population counts serving as the covariate. Abamectin had moderate impact and fluopyram had substantial impact on the non-target nematodes. Furfural and fluensulfone had minimal impact on non-target nematodes. The results of this study suggest nematicides can impact non-target nematode densities in bermudagrass.

Turfgrass (*Cynodon* spp.) is an important horticultural crop in the Southeastern United States. Golf courses, athletic fields, and lawns utilize turfgrass as a playing surface and as ground cover. Turfgrass cultivation, sales, and maintenance is a billion-dollar industry in Florida (Haydu et al., 2006). Plant-parasitic nematodes are an important pathogen of turfgrass. Nematode feeding can lead to stunted roots and even death of the plant (Crow, 2008). As an aesthetic crop, turfgrass managers typically have low thresholds for damage. Chemical nematicides are valuable for managing injurious nematode populations, particularly on golf courses (Crow et al., 2003). Nematicides have been shown to reduce plant-parasitic nematodes, but few studies have evaluated the effect on non-parasitic nematodes. These include bacterial-feeding (bacterivores), fungal-feeding (fungivores), omnivorous (omnivores), and predaceous (predatory) nematodes. Because of the high abundance, ubiquitous nature, and occupancy of many trophic levels, nematodes have been used as bioindicators for ecological studies in regard to soil condition (Zullini and Peretti, 1986; Bernard, 1992; Yeates et al., 1993; Neher and Campbell, 1994; Bongers and Ferris, 1999). Nematode groupings are typically at the family level and reflect life-history strategy as colonizers or persisters (cp) in the soil ecosystem (Ferris et al., 2001). Nematode functional group analysis can be performed by comparing shifts in community structure of different feeding groups or by using more advanced metrics. Diverse soil community structure contains members at different trophic levels. Some nematodes affect nutrient cycling by contributing to steady microbial growth from grazing on bacteria or fungi and others may help suppress plant-parasitic nematode numbers through predation (Dindal, 1990). Alterations in soil community structure may affect ecosystem health by altering the ability of soil to function as a living system (Doran and Zeiss, 2000; Ferris and Bongers, 2006).

Studying population changes of nematode functional groups can provide insight into potential effects of nematicides on soil health (Ferris et al., 2001). We conducted nematicide treatment programs in order to better understand potential non-target effects on non-herbivore free-living nematode community structure. We predicted nematicide applications would significantly affect nematode community structure by decreasing the number of nematodes belonging to high trophic levels and increased abundance from low trophic level nematodes.

## Material and methods

### Study site

Studies were conducted at the University of Florida Plant Science Research Unit (PSU) in Citra, FL. The study field was planted with ‘Tifdwarf’ bermudagrass and maintained with typical turfgrass management practices by the staff at PSU. The only chemicals used for maintenance were fertilizer, plant growth regulator, and herbicides. The field was treated as needed with thiencarbazone-methyl, foramsulfuron and halosulfuron-methyl, sulfentrazone, and trinexapac-ethyl for weed control and turf management. Plots were fertilized with a 13-4-13 controlled-release golf course green fertilizer during the growing season. Soil was 97% sand, 1% silt, 2% clay; 4% organic matter; pH 7.1.

### Treatment applications

The experiment used a randomized block design with five treatments and five replicates. In addition to an untreated control, the experimental treatments used were: abamectin (Divanem; Syngenta Crop Protection, Raleigh, NC), furfural (MultiGuard EC; Agriguard, Cranford, NJ), fluopyram (Indemnify; Bayer CropScience, Raleigh, NC), and fluensulfone (Nimitz Pro G; ADAMA Agricultural Solutions, Tel Aviv, Israel). Rates were based on the maximum allowable rate as listed on each label (Table 1).

**Table 1:** Nematicide formulations used in the field study and their per-application labeled application rates.

Active Ingredient (a.i.)	Trade name	Application rate	Formulation
Abamectin	Divanem	0.89 liters product/ha (70 g a.i./ha)	EC^a^
Fluopyram	Indemnify	1.25 liters product/ha (500 g a.i./ha)	EC
Furfural	MultiGuard Protect	56 liters product/ha (60 kg a.i./ha) 2016^b^ 74 liters product/ha (77 kg a.i./ha) 2017	EC
Fluensulfone	Nimitiz Pro G	62.25 kg product/ha (1 kg a.i./ha)	G

^a^EC, emulsifiable concentrate; G, granular. ^b^Labeled rate changed between 2016 and 2017.

Applications of liquid treatments were made using a CO_2_-powered backpack sprayer (Weed Systems, Hawthorne, FL) with TJ-08 nozzles delivering 1,222 liters solution/ha. Nimitz Pro G was applied using a walk-behind Gandy (Owatonna, MN) drop-spreader. Plots were 6 m^2^ with 1.5 m^2^ data collection plots located in the center of each larger treatment plot to minimize any cross-contamination between plots. All plots were separated by an untreated 0.6 m border on each side. After each application, all treated and untreated plots were immediately irrigated with 0.64 cm of water. Treatments were applied every four weeks replicating a summer treatment program from June 7 to August 30 in 2016 and April 24 to July 18 in 2017.

### Sampling

Samples were collected prior to the initial treatment, and 2 d, 14 d, 56 d, and 238 d after the final treatment application (DAFT) each year. Turf plugs were collected using a 3.81-cm diameter ball mark plugger (Turf-Tec International, Tallahassee, FL) to a depth of 6.35 cm. Eight plugs were collected from the data collection subplots and combined in plastic sampling bags for analysis. The soil was shaken from the thatch and roots and nematodes were extracted from 100 cm^3^ of this soil by centrifugal flotation (Jenkins, 1964). Of the thatch and roots from the eight plugs, four were used for extraction of nematodes using mist extraction (Seinhorst, 1950). Turf plug extraction was performed separately from soil to target nematodes inhabiting the thatch layer that are not extracted as efficiently from soil (Crow, 2017). The remaining four plugs were used for arthropod extraction as part of a separate experiment. The misting chamber was a rectangular plexiglass structure containing PVC pipe running the length of the top plexiglass panel with misting nozzles arranged to provide a downward mist spray on the turf plugs. Nozzles were spaced 40 cm lengthwise and 30 cm widthwise along the length of the chamber. Funnels were placed 68 cm below nozzles in holes cut in a sheet of plexiglass to support each funnel. A mesh screen was placed on top of the funnel to support the turfgrass plugs. Mesh holes were 2 × 1 mm. Mist was sprayed on the samples for 45 sec every hour controlled by a solenoid valve (Hunter Industries, San Marcos, California) set on a recycling timer (Hydrofarm, Petaluma, California). Samples were collected in an Erlenmeyer flask below each funnel. Turfgrass plugs were left in the misting chamber for 72 hr and the collected specimens were preserved in 2% formalin and stored in plastic centrifuge tubes. Nematodes were identified morphologically and counted from soil and mist extraction samples using an inverted microscope (Olympus Corporation, Shinjuku, Tokyo, Japan). The primary guides used for nematode identification were Smart and Nguyen (1985) and Bongers (1988).

### Data collection

Data plots were photographed every two weeks using a digital camera mounted on a custom-built photo box throughout the growing period and continued until grass dormancy in the winter. Digital images were taken in center of data plots to be analyzed for the number of green pixels (hue 45–105, saturation 15–100) present in each image as a measure of turfgrass health using the macro developed by Karcher and Richardson (2005). Nematodes collected by mist and soil extraction were counted in gridded counting dishes using inverted microscopes. In total, 100 nematodes were identified from each sample, or the entire sample if the total number was fewer, to the family level.

### Statistical analysis

Population counts were analyzed using analysis of covariance (ANCOVA) using R software version 3.3.2 (R Core Team, 2016). Data were log transformed to improve normality and homogeneity of variance. Population means of the different sampling dates were compared to the initial sample means using the untreated control as a covariate. ANCOVA was chosen to help account for natural seasonable variation. Nematode families were grouped into the feeding groups proposed by Yeates et al. (1993). Groups included were bacterivores, fungivores, herbivores, omnivores, and predators. The relative abundance of each feeding group was evaluated throughout the course of the two-year study. Data generated from both extraction methods were compared using a *t*-test at each sampling date to determine if extraction method had a significant (*P*⩽0.1) effect on the number of nematodes extracted. Additional analyses of ecological indices were performed by the Nematode Indicator Joint Analysis software developed by Sieriebriennikov et al. (2014). The nematode ecological indices used in this study were: maturity index (MI), enrichment index (EI), structure index (SI), and faunal profile based on the works of Bongers (1990), Ferris et al. (2001), and Ferris and Bongers (2009). Faunal profile was different from the untreated control if both enrichment index and structure index were determined to be different from the untreated control using the analysis of covariance. Because faunal profile incorporates EI and SI, the results from those indices are not shown separately.

## Results

In total, 24 nematode families were identified during the study. Of the families encountered, 6 families were categorized as plant-parasitic and 18 were categorized free-living. Nine of the families were bacterial-feeding, three were fungal-feeding, one was omnivorous, and five were predatory (Table 2). The bacterial-feeding family Cephalobidae was the dominant family representing 30% of all nematodes across both extraction processes. The dominant fungal-feeding family was Tylenchidae making up 18% of nematodes. Hoplolaimidae was the most abundant plant-parasitic group at 15% of total nematodes which was dominated by *Helicotylenchus* spp. nematodes. The omnivore family Aporcelaimidae was fourth most abundant at 13%. Data collected from each sampling method are presented separately since a significant effect of extraction method on nematode counts was observed (*P*⩽0.05).

**Table 2:** Nematode families identified from turfgrass plugs and soil samples.

Family	Cp value	Proportion of total mist extracted nematodes	Proportion of total soil extracted nematodes	Proportion of total nematodes
*Bacterivores*				
Bathyodontidae	4	<0.01	<0.01	<0.01
Cephalobidae	2	0.27	0.33	0.30
Diplogasteridae	1	<0.01	<0.01	<0.01
Diploscapteridae	1	<0.01	<0.01	<0.01
Diphtherophoridae	3	<0.01	<0.01	<0.01
Monhysteridae	2	<0.01	<0.01	<0.01
Plectidae	2	0.01	<0.01	0.01
Rhabditidae	1	<0.01	0.01	0.01
Teratocephalidae	3	<0.01	0.01	0.01
*Fungivores*				
Anguinidae	2	<0.01	<0.01	<0.01
Aphelenchidae	2	0.05	0.02	0.03
Tylenchidae	2	0.28	0.10	0.18
*Omnivores*				
Aporcelaimidae	5	0.08	0.17	0.13
*Predators*				
Discolaimidae	5	<0.01	<0.01	<0.01
Ironidae	4	<0.01	<0.01	<0.01
Monochidae	4	0.00	<0.01	<0.01
Qudsianematidae	4	0.08	0.02	0.04
Thornenematidae	5	<0.01	0.01	0.01
*Herbivores*				
Belonolaimidae	3	<0.01	0.02	0.03
Criconematidae	3	<0.01	0.01	0.01
Heteroderidae	3	0.10	0.05	0.08
Hoplolaimidae	3	0.08	0.20	0.15
Longidoridae	5	0.02	<0.01	0.01
Trichodoridae	4	<0.01	<0.01	<0.01

### Bacterivores

**Figure 1:**
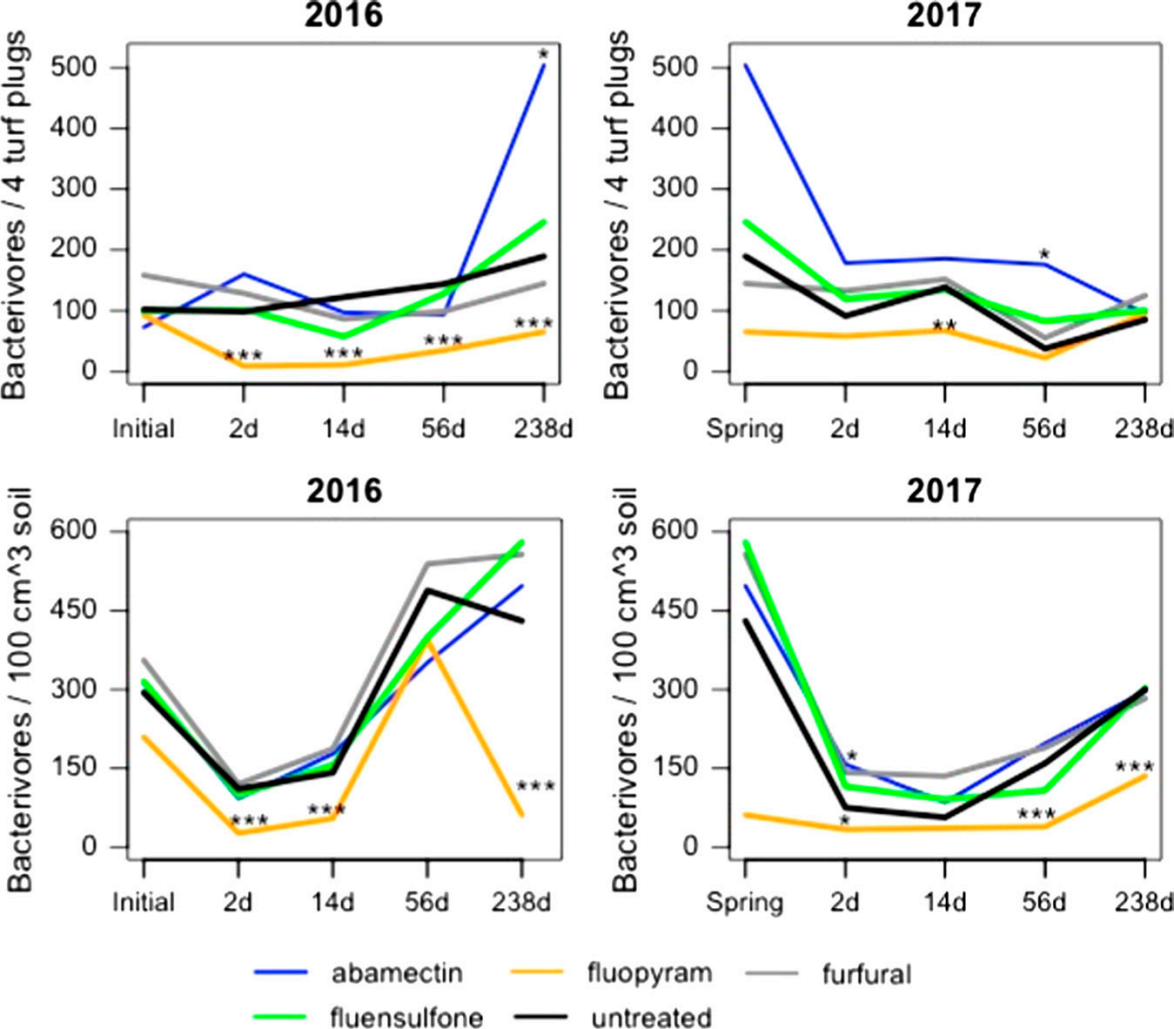
Population densities of bacterivore nematodes from mist and soil extraction as affected by different nematicide applications at all sampling dates. *,**,***Different from the untreated according to analysis of covariance, *P*⩽0.1, 0.05, and 0.01, respectively.

Bacterivores in abamectin-treated plots increased at 238 d after final treatment (DAFT) relative to the untreated control (*P*⩽0.1) in 2016 from mist extraction (ME) (Fig. 1). Bacterivores increased in abamectin-treated plots at 56 DAFT from ME and 2 DAFT from soil extraction (SE) relative to the untreated control (*P*⩽0.1) in 2017 (Fig. 1). Fluopyram decreased bacterivores relative to the untreated control at 2, 14, 56, and 238 DAFT in 2016 from ME (*P*⩽0.01) (Fig. 1). Fluopyram reduced bacterivore abundance at 2, 14, and 238 DAFT in 2016 from SE (Fig. 1). Fluopyram decreased bacterivores 14 DAFT relative to the untreated control from ME in 2017 (*P*⩽0.05) (Fig. 1). Numbers of bacterivores were reduced (*P*⩽0.1) by fluopyram 2, 56, and 238 DAFT relative to the untreated control from SE in 2017 (Fig. 1).

### Fungivores

**Figure 2:**
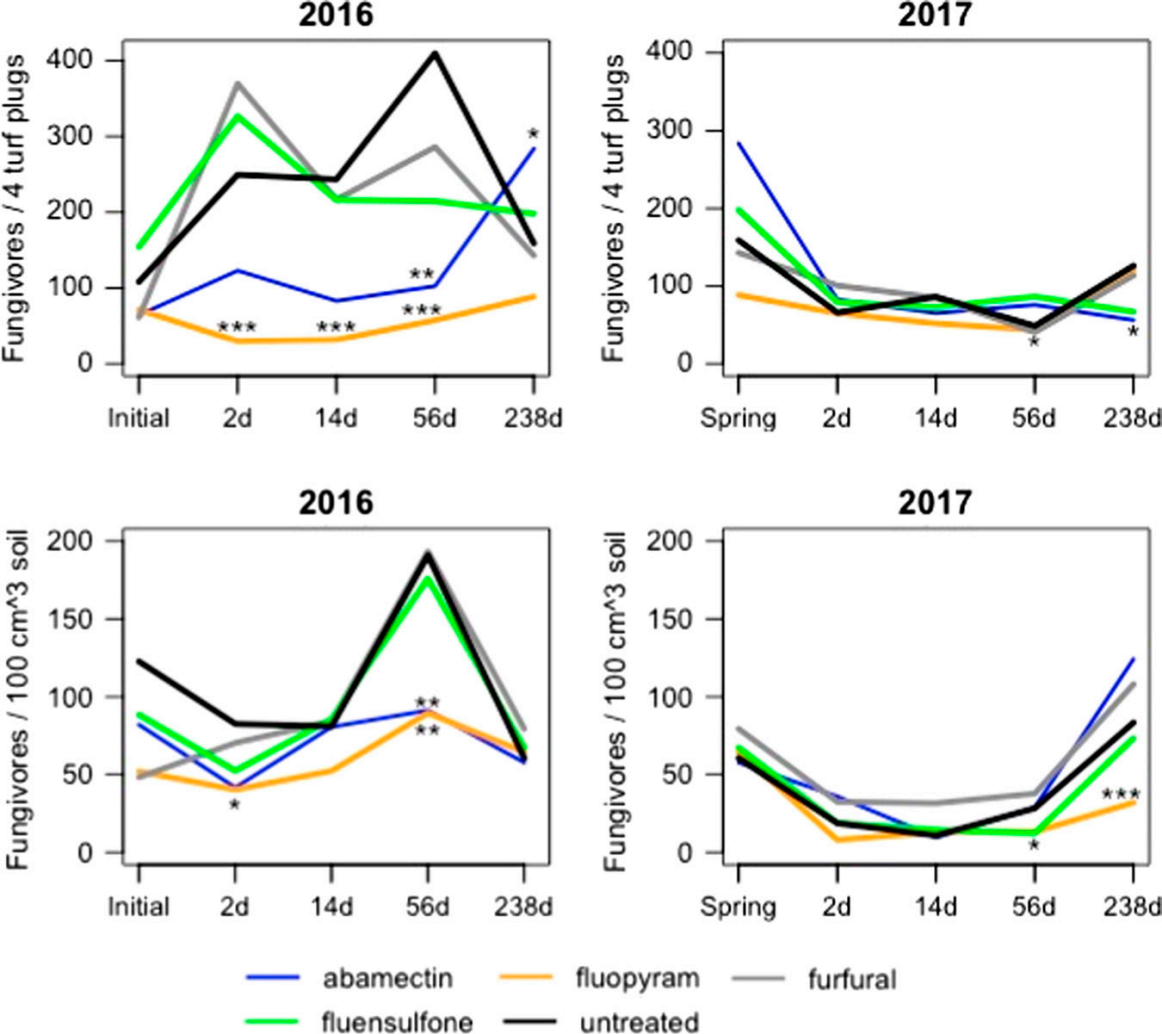
Population densities of fungivore nematodes from mist and soil extraction as affected by different nematicide applications at all sampling dates. *,**,***Different from the untreated according to analysis of covariance, *P*⩽0.1, 0.05, and 0.01, respectively.

Abamectin-treated plots had reductions in fungivores (*P*⩽0.05) relative to the untreated control at 56 DAFT in 2016 from ME and SE (Fig. 2). Fungivore abundance increased in abamectin plots (*P*⩽0.1) relative to the untreated control at 238 DAFT from ME in 2016 (Fig. 2). Abamectin reduced fungivore abundance relative to the untreated control at 238 DAFT from ME in 2017 (Fig. 2). Fluopyram reduced fungivores (*P*⩽0.01) relative to the untreated control at 2, 14, and 56 DAFT in 2016 from ME (Fig. 2). Fungivore abundance decreased in fluopyram-treated plots at 2 and 56 DAFT relative to the untreated control (*P*⩽0.1) in 2016 from SE (Fig. 2). Fluopyram reduced fungivore abundance relative to the untreated control 238 DAFT from SE in 2017 (Fig. 2). Furfural reduced fungivore abundance relative to the untreated control 56 DAFT from ME in 2017 (Fig. 2). Fluensulfone-treated plots had fewer fungivores relative to the untreated control (*P*⩽0.1) 56 DAFT from SE in 2017 (Fig. 2).

### Omnivores

**Figure 3:**
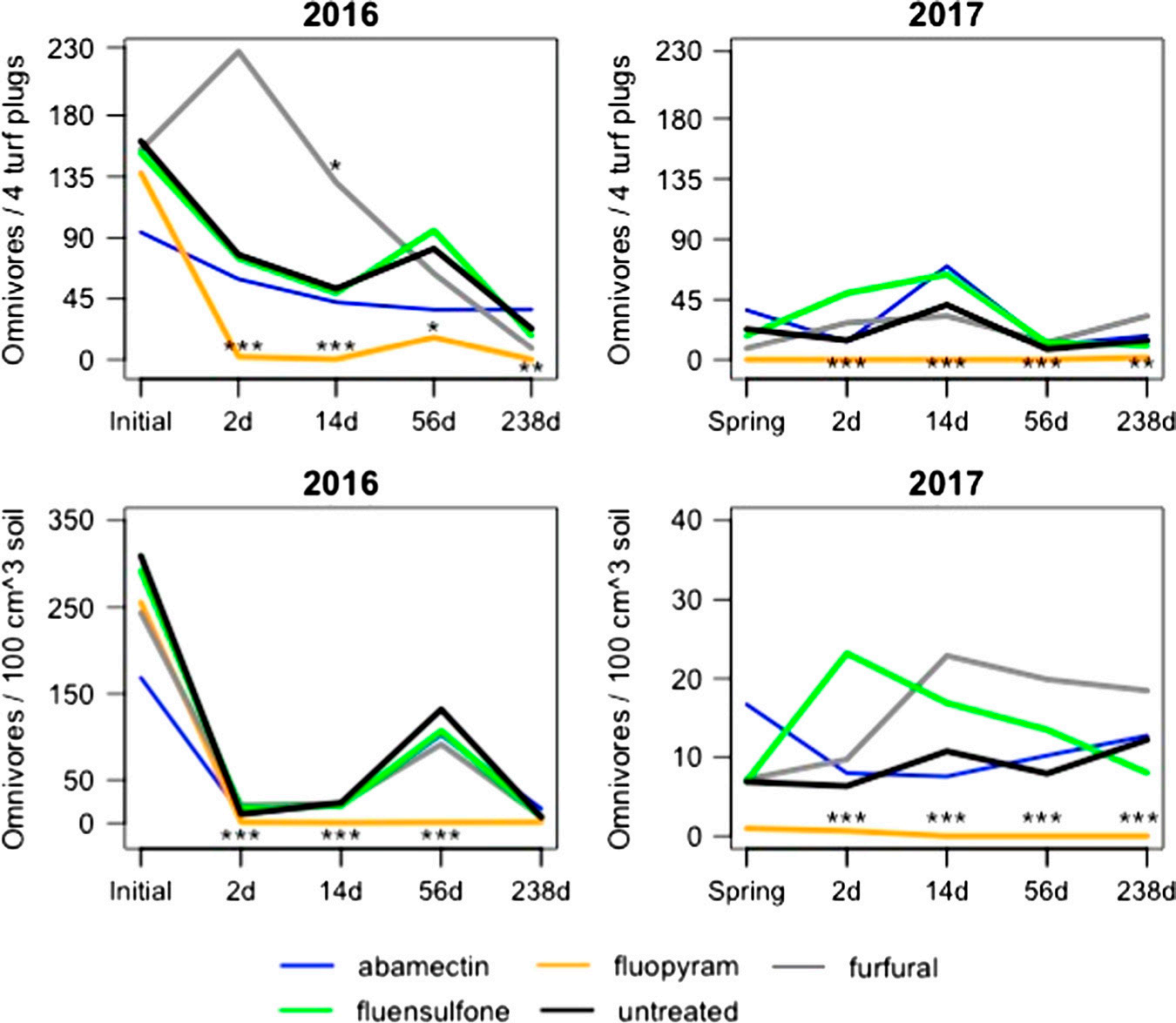
Population densities of omnivore nematodes from mist and soil extraction as affected by different nematicide applications at all sampling dates. The scale of the y-axis for soil extraction is different for 2017 than for 2016. *,**,***Different from the untreated according to analysis of covariance, *P*⩽0.1, 0.05, and 0.01, respectively.

Fluopyram reduced omnivore population densities relative to the untreated control (*P*⩽0.1) at 2, 14, 56, and 238 DAFT in 2016 from ME and reduced population densities relative to the untreated control (*P*⩽0.01) at 2, 14, and 56 DAFT from SE in 2016 (Fig. 3). Omnivore abundance in fluopyram-treated plots was also lower relative to the untreated control (*P*⩽0.05) at 2, 14, 56, and 238 DAFT from ME and SE in 2017 (Fig. 3). Furfural plots had increased omnivores relative to the untreated control (*P*⩽0.1) 14 DAFT from ME in 2016 (Fig. 3).

### Predators

**Figure 4:**
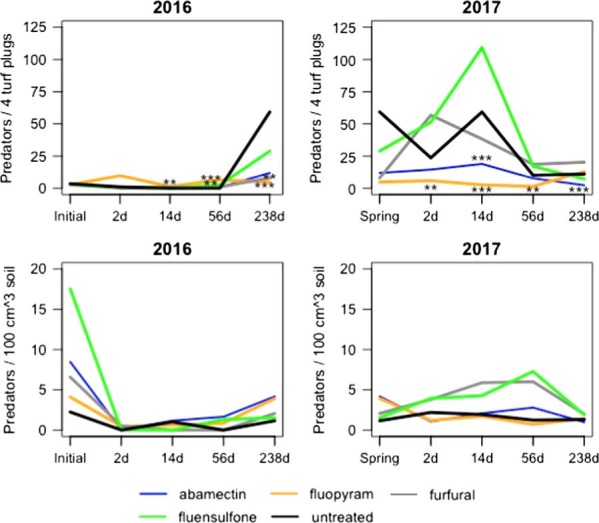
Population densities of predatory nematodes from mist and soil extraction as affected by different nematicide applications at all sampling dates. *,**,***Different from the untreated according to analysis of covariance, *P*⩽0.1, 0.05, and 0.01, respectively.

Abamectin reduced predator abundance relative to the untreated control (*P*⩽0.01) at 14 and 238 DAFT from ME in 2017 (Fig. 4). Fluopyram-treated plots had greater predator abundance relative to the untreated control at 14 and 56 DAFT from ME (*P*⩽0.01) in 2016 and decreased population densities relative to the untreated control (*P*⩽0.01) at 238 DAFT from ME in 2016 (Fig. 4). Fluopyram decreased predators relative to the untreated control (*P*⩽0.05) at 2, 14, and 56 DAFT from ME in 2017 (Fig. 4). Furfural applications decreased predator abundance relative to the untreated control (*P*⩽0.05) at 238 DAFT from ME in 2016 (Fig. 4). Fluensulfone-treated plots had increased predator abundance relative to the untreated control (*P*⩽0.05) at 56 DAFT from ME in 2016 (Fig. 4).

### MI

**Figure 5:**
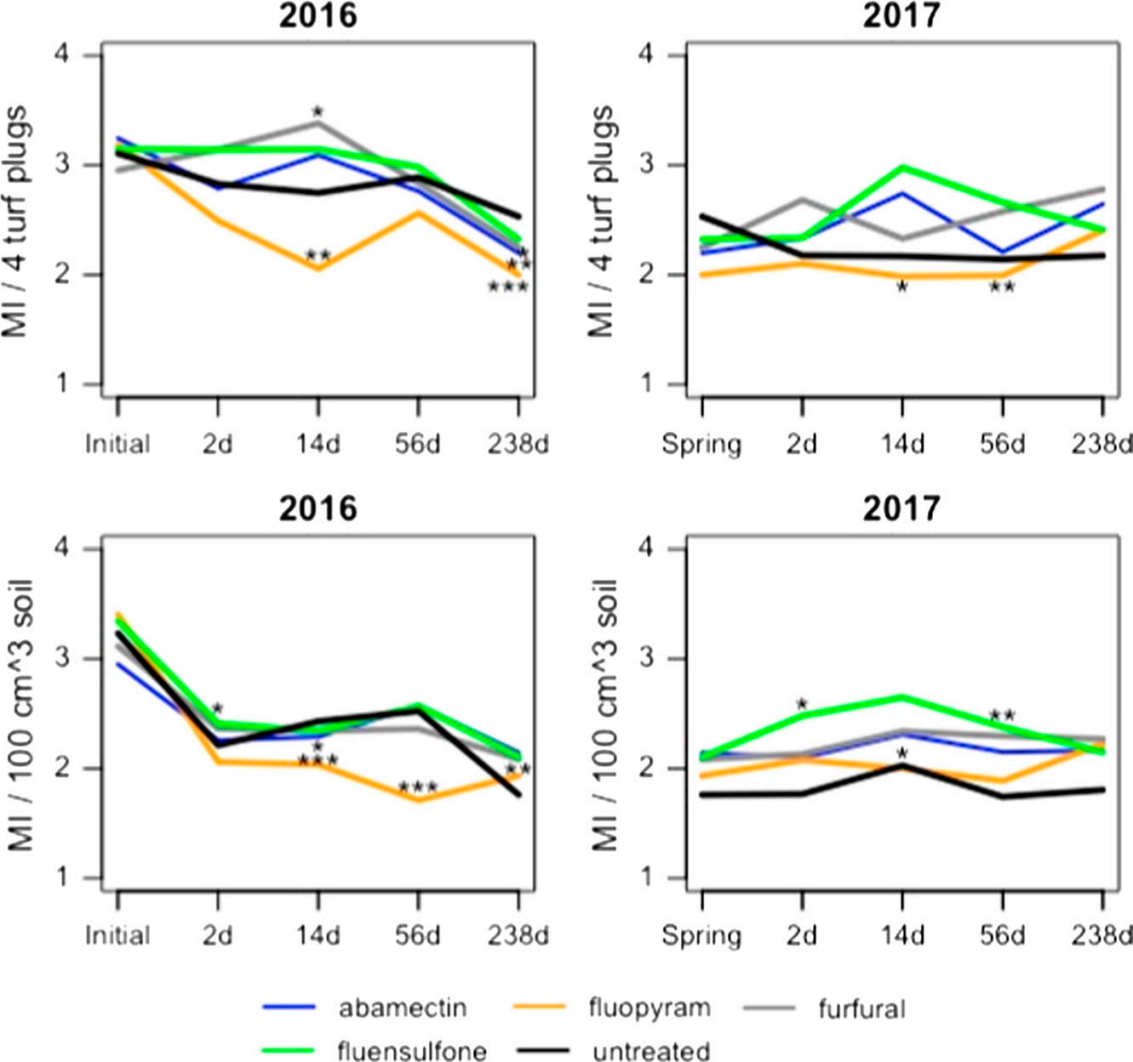
Maturity index (MI) from mist and soil extraction as affected by different nematicide applications at all sampling dates. *,**,***Different from the untreated according to analysis of covariance, *P*⩽0.1, 0.05, and 0.01, respectively.

Abamectin lowered MI relative to the untreated control (*P*⩽0.1) at 14 DAFT from SE and 238 DAFT from ME in 2016 (Fig. 5). Fluopyram reduced MI relative to the untreated control (*P*⩽0.05) at 14 and 56 DAFT from SE and at 14 and 238 DAFT from ME in 2016 (Fig. 5). MI increased relative to the untreated control (*P*⩽0.05) in fluopyram-treated plots at 238 DAFT from SE in 2016 (Fig. 5). Fluopyram reduced MI at 14 and 56 DAFT relative to the untreated control (*P*⩽0.1) from ME and at 14 DAFT from SE in 2017 (Fig. 5). Furfural treatments resulted in increased MI relative to the untreated control (*P*⩽0.1) at 14 DAFT and decreased relative to the untreated control (*P*⩽0.1) at 238 DAFT from ME in 2016 (Fig. 5). Fluensulfone-treated plots had increased MI relative to the untreated control (*P*⩽0.1) at 2 DAFT from SE in 2016 and at 2 and 56 DAFT relative to the untreated control (*P*⩽0.1) from SE in 2017 (Fig. 5).

### EI and SI

Abamectin-treated plots had significant reduction in EI compared to the untreated control (*P*⩽0.05) from ME at 14 DAFT in 2016 and at 238 DAFT 2017 (data not shown). Abamectin-treated plots had significant increase in EI compared to the untreated control (*P*⩽0.1) at 14 DAFT from SE in 2016. Furfural-treated plots had significant decrease in EI compared to the untreated control (*P*⩽0.05) at 238 DAFT from ME in 2017. Fluensulfone-treated plots had significant reduction in EI compared to untreated control (*P*⩽0.05) at 56 DAFT from SE in 2016 and at 238 DAFT from ME in 2017. Fluopyram-treated plots had significant increase in EI relative to the untreated control (*P*⩽0.05) at 238 DAFT from ME and at 14 and 238 DAFT from SE in 2016 (data not shown). Fluopyram-treated plots had significant reductions in SI relative to the untreated control (*P*⩽0.1) at 2, 14, and 238 DAFT from ME and significant reductions in SI relative to the untreated control (*P*⩽0.05) at 2, 14, and 56 DAFT from SE in 2016.

### Faunal profile

Data points from all plots prior to the first treatment were clustered in quadrats C and B from ME and all in quadrat C from SE. Data points migrated from quadrat C to being divided between quadrats D and C over the course of the study. Environmental conditions shifted from a highly structured, moderately enriched environment to a low structure, low enrichment environment. Enrichment and structure indices calculated from abamectin, furfural, and fluensulfone plots were not different from untreated control at the same date from either extraction method (*P*>0.1). Fluopyram-treated plots had lower structure and greater enrichment compared to the untreated control at 238 DAFT from ME (*P*⩽0.1) and from SE at 14 DAFT during 2016 (*P*⩽0.05) (data not shown).

### Percent green cover

Photograph data were analyzed from 38 time points across the two-year study (data not shown). All four nematicides significantly affected percent green coverage (*P*⩽0.1) at multiple times during the trial. Abamectin significantly impacted green coverage relative to the untreated control at 18 dates, fluopyram at 22 dates, furfural at 8 dates, and fluensulfone at 8 dates.

## Discussion

### Abamectin

Abamectin had intermediate effects on nematodes, altering the community structure of free-living nematodes and causing disturbance to the soil ecosystem as indicated by a shift to a slightly less mature soil food web. Effects on nematodes were generally detected later in the season and could be explained by the characteristic slow movement of this formulation through the thatch layer of turfgrass (Gannon et al., 2017). High cp nematodes were mildly affected by abamectin. The reduction in higher trophic nematodes at the later sampling dates was accompanied by bacterivore increases at a few dates; possibly influencing the trophic cascade by reducing pressure of predatory nematodes on bacterivores (Wardle and Yeates, 1993). The enrichment index did not indicate an enriched environment; however, this index considers both bacterivore and fungivore abundance and could be offset by the reduction in fungivores belonging to the cp-2 class. Maturity index and structure index values suggested abamectin plots had a basal environment with reduced abundance of high trophic nematodes at the end of the study. Abamectin had a positive effect on turfgrass health as measured by percent green coverage, which is an important consideration.

While the nematicidal properties of avermectin family members have been known for some time, few studies have considered the effects of abamectin or ivermectin on free-living nematodes (Brinke et al., 2010; Bai and Ogbourne, 2016). Ivermectin contained in feces from cattle treated for animal-parasitic nematodes has been shown to affect *Tylenchus* and *Cephalobus* free-living nematodes in pastureland (Yeates et al., 2003). Farmland treated with pesticides including abamectin has been shown to undergo moderate disturbance based on maturity indices, plant-parasitic index, fungivore/bacterivore abundance, basal index and channel index evaluations (Dong et al., 2008). A study on freshwater nematodes found free-living nematode abundances were impacted by the exposure of ivermectin (Brinke et al., 2010).

### Fluopyram

Fluopyram had the most striking results of the nematicides tested. It reduced numbers of both beneficial nematode and plant-parasitic nematodes at intermediate and long-term sampling intervals in both years. Bacterivores, fungivores, and omnivores were susceptible to fluopyram at most sampling dates and predators were reduced at intermediate and late sampling dates. These observations suggest fluopyram has the potential to affect all nematode feeding groups quickly after application and throughout the season. Fungal feeders were not significantly affected. EI values revealed an enriched environment after fluopyram application on a couple dates. In addition to the reduction of nematode functional group densities, a shift occurred toward an environment dominated by *r* strategist nematodes. The soil condition shifted toward a more basal and degraded environment. Fewer trophic linkages in the nematode food web existed compared to the untreated control based on maturity index and structure index. This environment is less stable and would likely be more prone to reductions in ecosystem service benefits.

### Furfural

Furfural had a low impact on free-living nematodes. The fungivore and predatory nematode functional groups were the only feeding groups negatively affected by furfural. The general lack of reduction in functional group densities suggests furfural may have low risk to free-living nematodes. No adverse effects on free-living nematodes were observed, which support results obtained by Abdelnabby et al. (2016, 2018), although negative impacts on bacterivore and fungivore nematodes from furfural have been documented in tomato field trials (Ntalli et al., 2018). Plant-parasitic nematodes increased at two-week sampling dates in one year (data not shown). These results may be attributed with temporary *Meloidogyne* spp. increases occasionally seen with furfural use (Crow and Luc, 2014). The gelatinous matrix of *Meloidogyne* spp. egg masses is thought to be dissolved by furfural releasing juveniles into the soil (Steyn and Van Vuuren, 2006). Soil condition was found to be stable based on maturity indices. Furfural had a moderate impact on turfgrass green cover. Positive responses in turf health were observed in both years, but generally after the beginning of September. Lack of furfural efficacy in the top 5 cm of soil has been observed in other trials (Crow and Luc, 2014). As a byproduct from sugarcane processing, furfural is readily broken down by microbes. It has been hypothesized that in field conditions furfural is broken down before activity on nematodes occurs. This could contribute to the minimal effects on functional groups observed in our study.

### Fluensulfone

Fluensulfone had low impacts on free-living nematodes comparable to furfural. Fungivores were the only functional group negatively affected at one sampling date in 2017. Predators increased in abundance at eight weeks in both years. As a result, structure and food web complexity were greater in fluensulfone-treated plots than untreated control according to the maturity indices and SI. Laboratory-based assays have found the plant-parasite *M. javanica* to be susceptible to fluensulfone at lower doses than the bacterivore *C. elegans* (Kearn et al., 2014). This compound has a mode of action distinct from the more traditional organophosphate, carbamate, and ivermectin active ingredients potentially having a lower impact on the soil ecosystem. Fluensulfone also has systemic activity which could allow for plant-parasitic nematode control in plant roots even if the compound has moved past the rhizosphere. Low toxicity to earthworms has been documented, but little is known about the impact of fluensulfone on other soil fauna (Oka et al., 2012; Beknazarova et al., 2016). The low risk perceived to non-target soil fauna has led to its promotion as an environmental control measure against soilborne stages of the human parasite *Strongyloides* (Beknazarova et al., 2016). Another factor that may have led to limited effects on non-target invertebrates is persistence; the manufacturer reports a short half-life of 7 to 17 d in soil and the formulation used in the study has rapid movement through the thatch and soil profile (Oka et al., 2013; Crow et al., 2017). Short exposure time and reduced toxicity to non-target nematodes could contribute to the low impact on free-living nematodes. The results observed support the claim fluensulfone has a low risk to free-living nematodes.

### Summary

In summary, nematicides can negatively impact free-living nematodes in bermudagrass. Fluopyram had the greatest impacts on nematode functional groups followed by abamectin in this study. Despite the negative effects on free-living nematodes, turfgrass percent green cover was generally higher in both fluopyram and abamectin plots. Furfural and fluensulfone had low impacts on free-living nematodes, but low impacts on turfgrass green cover were also observed. Nematicides are used to reduce the impact of plant-parasitic nematodes and it is, therefore, expected that nematicides with greater efficacy tend to have greater impact on nematode community structure.

Our hypothesis that “nematicide applications would significantly affect nematode community structure by decreasing the number of nematodes belonging to high trophic levels and increased abundance from low trophic level nematodes” was only partially supported. Our results show that the nematicides with the greatest impact, fluopyram, reduced numbers of both high and low cp nematodes. Therefore, the effects of nematicides on faunal profile were minimal because all trophic groups were impacted similarly.

While there were significant impacts from the treatments, it should not be assumed that they were all due to direct effects of the chemicals. For example, some treatments resulted in healthier grass than other treatments, and plant health can drive an ecosystem. Similarly, the treatments might affect arthropods, fungi, bacteria, etc., that are predators, pathogens, or food for different types of nematodes and influence the nematode community structure that way. Finally, while the treatments were all applied according to their labels, their application rates and timing may not reflect how they would be recommended for use in the field. For research purposes, they were all applied on the same schedule using the maximum rate allowable. However, a golf course might make fewer applications, space out treatments at different times of the year, apply lower rates than the maximum, or rotate chemistries. The objective of this research was not to indicate that certain nematicides were better than others. Rather, the intent was to introduce the concept of soil ecosystem health into the discussion of golf course nematode management and to promote further research into nematode integrated pest management.
